# Do complexity-informed health interventions work? A scoping review

**DOI:** 10.1186/s13012-016-0492-5

**Published:** 2016-09-20

**Authors:** Julii Brainard, Paul R. Hunter

**Affiliations:** Norwich Medical School, University of East Anglia, Norwich, NR4 7TJ UK

**Keywords:** Complex adaptive systems, Complexity science, Scoping review, Interventions, Health service

## Abstract

**Background:**

The lens of complexity theory is widely advocated to improve health care delivery. However, empirical evidence that this lens has been useful in designing health care remains elusive. This review assesses whether it is possible to reliably capture evidence for efficacy in results or process within interventions that were informed by complexity science and closely related conceptual frameworks.

**Methods:**

Systematic searches of scientific and grey literature were undertaken in late 2015/early 2016. Titles and abstracts were screened for interventions (A) delivered by the health services, (B) that explicitly stated that complexity science provided theoretical underpinning, and (C) also reported specific outcomes. Outcomes had to relate to changes in actual practice, service delivery or patient clinical indicators. Data extraction and detailed analysis was undertaken for studies in three developed countries: Canada, UK and USA. Data were extracted for intervention format, barriers encountered and quality aspects (thoroughness or possible biases) of evaluation and reporting.

**Results:**

From 5067 initial finds in scientific literature and 171 items in grey literature, 22 interventions described in 29 articles were selected. Most interventions relied on facilitating collaboration to find solutions to specific or general problems. Many outcomes were very positive. However, some outcomes were measured only subjectively, one intervention was designed with complexity theory in mind but did not reiterate this in subsequent evaluation and other interventions were credited as compatible with complexity science but reported no relevant theoretical underpinning. Articles often omitted discussion on implementation barriers or unintended consequences, which suggests that complexity theory was not widely used in evaluation.

**Conclusions:**

It is hard to establish cause and effect when attempting to leverage complex adaptive systems and perhaps even harder to reliably find evidence that confirms whether complexity-informed interventions are usually effective. While it is possible to show that interventions that are compatible with complexity science seem efficacious, it remains difficult to show that explicit planning with complexity in mind was particularly valuable. Recommendations are made to improve future evaluation reports, to establish a better evidence base about whether this conceptual framework is useful in intervention design and implementation.

**Electronic supplementary material:**

The online version of this article (doi:10.1186/s13012-016-0492-5) contains supplementary material, which is available to authorized users.

## Background

Many influential articles of the last 20 years promoted using the lens of complexity science to improve health care delivery [1–8]. Health care and health care delivery are said to be increasingly complex [4], with outcomes that are often unpredictable and can even be paradoxical. As a result, possible solutions to some public health issues are not well-suited to the otherwise gold standard test method for intervention research (randomized controlled trials) [2, 3, 8], due to the sheer complexity of inputs as well as diverse interpretations or relative importance of many inputs and outputs. Inconsistent inputs, ever-changing active agents and institutions, unforeseen relationships and consequences are common aspects of real-life public health problems. Nonetheless, recognizing the inherent properties of complex systems may suggest opportunities to design more effective health care delivery [1, 4, 7].

In this paper, we use “complexity science” as an umbrella term for a number of closely related concepts: complex systems, complexity theory, complex adaptive systems, systemic thinking, systems approach and closely related phrases. All these concepts may be useful for working within systems with these features (among others):

- Large number of elements, known and unknown.
- Rich, possibly nested or looping, and certainly overlapping networks, often with poorly understood relationships between elements or networks.
- Non-linearity, cause and effect are hard to follow; unintended consequences are normal.
- Emergence and/or self-organization: unplanned patterns or structures that arise from processes within or between elements. Not deliberate, yet tend to be self-perpetuating.
- A tendency to easily tip towards chaos and cascading sequences of events.
- Leverage points, where system outcomes can be most influenced, but never controlled.

The merits of a perspective informed by complexity science (CS) in public health appear to be strongly supported by pragmatic acceptance of the nature of real-life situations, and also a useful counter-balance to the weaknesses in reductionist perspectives, and potentially over-optimistic reliance on evidence-based medicine [1, 2, 4]. Much previous applied research used CS ideas to undertake process evaluation (without requiring that CS principles were part of intervention design), usually leading to recommendations for future improvements in implementation [9, 10]. However, subsequent impacts after implementing recommendations derived from using the lens of complexity theory are not widely published. Even if such follow-up impacts were available, it can be argued that the results would still not be conclusive: inherently, cause and effect are hard to show in complex systems [11]. Complexity science is thus stuck in an ironic paradox of appearing very useful for evaluation but with uncertain value if applied to inform implementation. It is argued that lack of empirical evidence discourages widespread adoption of complexity-informed research design [12, 13].

Previous reviews looked for efficacy of the CS strategy in intervention design or implementation by defining what are the key CS principles to follow in order to find opportunities for positive change (leverage). Intervention programmes were scored for how well they seemed to follow these key principles, leading to some comments on final efficacy [14–17]. Other discussions concentrated on showing the impact of complexity-informed interventions at intermediate points in the possible transition to change—evidencing influence on the likely causal pathway without attempting to assess final impacts [8, 18]. These previous articles did *not* require that selected studies include phrases such as “complexity science” (or closely related terms). It was tacitly understood that many interventions which utilize ideas that are compatible with CS are not described as such. Among the few reviews that explicitly looked for any conceptual framework in intervention designs, Adam et al. [9] reviewed studies in low- and middle-income countries. Applying customized evaluation criteria, Adam et al. found that theoretical underpinning was often not described, and CS especially was often missing from design as well as process evaluation.

Our scoping review directly addresses the feasibility of and difficulties in finding elusive empirical evidence in complexity-informed interventions: is it possible to show that interventions purposefully designed with complexity concepts are effective? We then develop recommendations for both those seeking such evidence and those planning intervention research using a complexity-informed conceptual framework.

Hence, in this paper, we looked for cases where authors *explicitly* state that CS informed intervention design, and we also noted what evaluation metrics were used. However, interventions that utilized CS only as part of evaluation (and not in design) were excluded. In so doing, we attempt to explore some of the difficulties in finding, reporting on and evaluating interventions that have a design informed by CS. The approach is that of a scoping review because the core objective is not thorough assessment of correct application of complexity theory or rigorous assessment of final impacts (such as a systematic review [19]), but rather the viability of trying to find out if theory has been used purposefully, if reporting is useful to others, and how feasible is it to find evidence to demonstrate that CS-informed interventions lead to benefits.

## Methods

In late December 2015, a literature search was undertaken for relatively modern articles (published after 1994) using Scopus and Ovid bibliographic databases. Review documents were hand-searched, and supplemental searches performed on specific websites. A grey literature search was undertaken using English language search phrases. All search strategies are described in Additional file 1. Titles and abstracts were screened (single reviewer) for eligibility, and exclusion criteria were applied as below.

### Exclusions

Conference abstracts, editorials and poster presentations were excluded. Reports on education or professional development programmes were also excluded, unless they described changes in service delivery measures or patient outcomes (see “Eligibility” section). When a pilot study with limited evaluation or protocol was found that described a CS-informed intervention, follow-up evaluation reports were sought and considered for eligibility. Review papers were excluded but also hand-searched for eligible studies not previously found. There were no initial exclusions for country or language, but data extraction and detailed analysis (see below) was only undertaken on studies in the most common countries: UK, Canada and USA, in order to consider only relatively similar cultural contexts and health systems together, and because a similar and detailed evaluation of complexity-informed interventions was previously and recently undertaken for interventions in low- and middle-income countries [9].

### Eligibility

Interventions were only included where CS was explicitly stated as having influenced the intervention design (including implementation and delivery, but not solely in evaluation). Evaluation of impacts must also be present after implementation. A CS-informed design was indicated by the use of one of the following phrases or very similar wording, in the abstract or in the description of intervention design or theory underpinning intervention design:

#### Complex systems, complexity theory, complex adaptive systems, systemic thinking and systems approach

Several phrases that are often linked to complexity science were not by themselves adequate to confirm eligibility. These include the phrases (each citation indicates distinctions from applications using complexity science) “systems change” [20], “complex interventions” [6], “systems science” [21] and “quality improvement” [22]. Instead, CS had to be explicitly cited using one of the search phrases as part of the conceptual framework for intervention design.

The evaluation must include at least one observed change that was believed by the authors to be linked or possibly linked to the intervention, and the evaluation metric had to be specific, not a simple opinion that the intervention had been helpful or increased confidence, but rather helpful in a specific way or context. A statement such as “I feel more confident” was inadequately specific and thus excluded, but the statement “I am now more confident *when contributing to meetings*” would be included. Changes were typically reported for perspectives, habits, service performance indicators, clinical or treatment targets and/or patient outcomes. However, the pathway between intervention and outcomes did not need to be highly detailed. Moreover, many interventions had multiple components (not only CS-informed), and the authors did not have to explain an explicit causal link between the CS-informed aspects of the intervention and any particular outcome.

After screening, we further focused on interventions in the three single countries which had yielded the most articles: USA, UK and Canada. Adam et al. [9] previously sought and evaluated complexity-informed theoretical underpinnings in interventions in low- and middle-income countries.

### Data extraction and thematic analysis

The approach of this review is necessarily more narrative and qualitative than quantitative, and extraction therefore tried to capture data that can be categorized thematically. Table 1 shows the data that were extracted from each included study, using a standardized form. The results were grouped thematically and are discussed narratively.

**Table 1:** Extracted data from each study

Bibliographic details
Which CS concept did authors use (exact phrase) How the intervention was implemented (e.g., workshops with health care professionals) What barriers were identified to implementation or achieving positive outcomes Time period (since intervention start) that observations were made for possible impacts Positive, negative or unintended consequences Were intervention agents (typically health professionals) consulted about unintended or negative consequences

### Quality assessment

Quality assessment was concerned with some aspects of evaluation, especially those relevant to complexity science. The concern was to determine whether reported results could be confidently linked to the intervention and, indeed, whether CS had informed evaluation as well as intervention design. Hence, the questions in Table 2 were asked about each intervention, where a “yes” answer was preferred and feasible to ascertain for each question. The justification for these questions is as follows: Complex systems are said to be hard to change; hence, changes should be observed over longer rather than shorter periods (Q1); evaluation should be consistent (Q2); were changes observed by impartial methods, or simply observed as opinion statements (Q3); poor response from participants was undesirable and could bias results (Q4); and a common feature of complex systems are unintended consequences following input changes; ideally, any evaluation report should look for them (Q5). Each quality assessment question was scored (−1 = No, 0 = partly or unclear, 1 = yes), and a composite (sum) score was generated for each study out of the five questions, to indicate quality of reporting. Interventions which scored 4 or 5 were interpreted as good, score = 3 interpreted as fair and others as less completely evaluated.

**Table 2:** Questions to assess quality of reporting

Q1. Were changes monitored for a period of at least 364 days after intervention start? Q2. Were outcomes measured in same way (all 3 of: units, measurement method, collection method) before & after implementation? Q3. Was the change measured objectively (vs. subjective statement, like “I make more referrals now”) Q4. Did a high percentage (≥80 % of sites or individuals) of intervention agents (e.g., health professionals or practice managers) respond to evaluation questions? Q5. Was there a stated methodology for looking for, or comment(s) by author(s) about observed unintended or negative consequences (which suggests they looked for them)	

## Results

Scientific literature searches were undertaken on 18–21 December 2015 and supplemental searches from 22 December 2015 to 4 January 2016. Three thousand four hundred sixty-four mostly unique scientific articles and 171 items from grey literature and reference checking were screened for eligibility and exclusion criteria. Grey literature search strategies and results are in Additional file 1. Sixty-six articles could not be excluded after screening abstracts and titles. At this stage, articles and reports came from countries: USA (*n* = 29, 44 %), UK (*n* = 9, 13.6 %), Canada (*n* = 5, 7.6 %), sub-Saharan Africa (*n* = 15, 22.7 %) and other parts of the world (Australia, Indian subcontinent, Taiwan, Morocco and Bosnia-Herzegonvina: *n* = 8, 12.1 %).

**Fig. 1:**
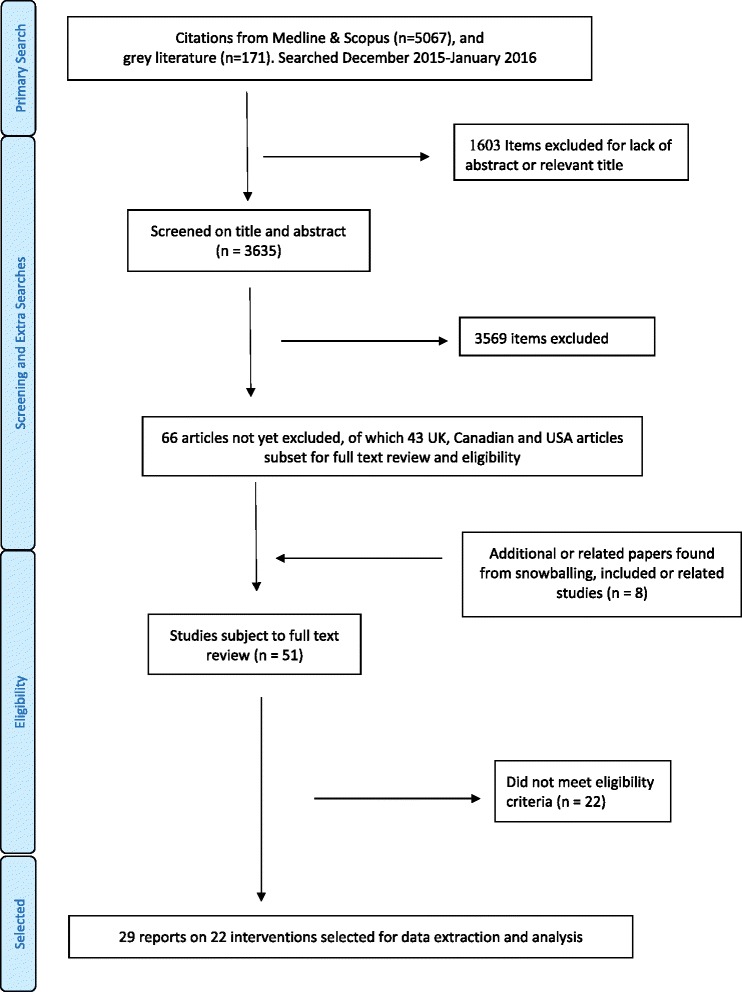
Study selection

Full-text review was confined to the 43 articles about interventions in the three most common high-income countries: Canada, UK and USA. Some interventions were reported in multiple articles. After full-text review, “snowballing” (reading text for references that seem to describe similar studies and checking those studies for references to similar studies), and looking for evaluations which followed promising protocols or pilot studies, the final selected and eligible interventions numbered 22, which were described in 29 reports. There were four interventions in the UK, three in Canada (described in five reports) and 15 interventions (described in 20 articles) in the USA. Figure 1 shows the study selection process. A list of included studies with key positive outcomes is in Table 3, while Additional file 2 gives further details on intervention format, complexity science concepts, timescale for observations, target conditions or problems and specific positive outcomes linked to each intervention.

**Table 3:** Selected studies, with examples of their positive impacts linked to interventions

Authors/name	Examples of positive outcomes
UK or Canada	
Chin et al. [38]	Better communication, more confidence in meetings or speaking to colleagues
Dattée et al. [39]	Change in mindset and practices from blame to mutual effort
Kothari et al. [40]	More positive feelings about collaborative practices
Mowles et al. [11]	Decrease in complaints, better staff retention, more focus in working practices
Rowe et al. [41]	More flexible working practices
Solomon et al. [42]	Referrals are quicker, more concise and appropriate
Zimmerman et al. [24], Gardam et al. [66], Gitterman et al. [23]	25–50 % falls in hospital acquired infections (MRSA and CDAD) at most participating hospitals
USA	
Backer et al. [44]	Some increases in breast cancer screening rates; extensive discussion of barriers
Balasubramanian et al. [27], Stroebel et al. [67]	Faster prescription refill; improved system-wide communication
Boustani et al. [46]	Fewer and shorter hospitalisations or visits to ER for dementia patients
Cabin Creek [35]	Fall from 400 to 30 narcotic prescriptions (=decline in unnecessary medication)
Capuano et al. [28]	Increased capacity in emergency dept., fewer diversions or transfer denials
Clancy et al. [43]	Calculated 64 % increase in efficiency, 9 sheets of paper saved per patient
Fontanesi et al. [29]	25 % increase in patient appointments, representing 9500 visits.
Horbar et al. [30]	Newborns received first dose of surfactant sooner compared to previous period (21 vs. 78 min post birth, *p* < 0.001).
Kegler et al. [25]	More collaborative working practices
Khan et al. [31]	Reduction in average brain function impediment score, drop from 19.21 to 14.75
MacKenzie et al. [32]	Turnaround time for beds reduced 210 to 54 min in 2004, maintained (2007)
Moody-Thomas et al. [33, 34]	Smoking quit rates increased from 5.0 % in 2008 to 9.6 % in 2009.
Parchman et al. [48, 49], Noël et al. [47]	Significant and sustained improvement in ACIC scores for chronic care: from 5.58 to 6.33 to 6.60 (group 1) or 5.27 to 5.99 (group 2), both *p* < 0.05.
Positive Deviance [36, 37]	Large falls in MRSA and other hospital acquired infections at many medical centres
Solberg et al. [45]	Diabetes management scores rose from 5.7 % to 42.9 %

### Applications

A distinction between UK/Canada and USA research emerged. All but one [23, 24] of the UK/Canadian reports had the objectives of changing mindsets and perspectives but without specific target outcomes. These types of interventions (also one in the USA [25]) could be described as primarily building personal competencies and capacities [26]. In contrast, the majority of US research (14 of 15 intervention programmes) identified specific targets linked to specific problems. These specific targets included increasing hospital capacity, improving neonatal care, improved critical care recovery, improved diabetes care, smoking reduction, cancer screening take-up, better medication management, improving dementia care, reduction in hospital acquired infections, improvements in chronic care indicators, and time savings in paperwork.

### Intervention format

Many interventions were implemented via a small number of consultative workshops or training sessions. Typically, in workshops (or retreats, or regular team meetings), a trained facilitator encouraged participants to identify and trial solutions to a specific problem [24, 27–37]. Audits, surveys, further reviews and feedback were often used to reinforce focus and objectives. The second most common approach was workshops designed to increase individual or group capacity across a range of health care responsibilities, with no specific target outcomes [11, 25, 38–42]. Three other applications of complexity science were employed for improvements to health care or service delivery. Clancy et al. [43] described a team of nurse managers who simplified care management forms, using the guideline that 80 % of cases fell into only 20 % of diagnoses and 20 % of cases comprised 80 % of diagnoses (power law tradeoff). Backer et al. [44] used complexity science to guide staff meetings which had the purpose of identifying barriers to imposition of care plans, so CS was used to facilitate implementation and identify barriers to a predesigned initiative. Solberg et al. [45] describe a single clinician applying systems thinking to identify, propose and test possible health care delivery improvements; team meetings facilitated this process, but the process was driven overwhelmingly by just one practitioner with a supervisory role.

### Barriers

Many studies did not describe any barriers to implementation [28, 30–32, 38, 43, 45, 46]. Difficulties in changing the larger health system and entrenched practices or habits of others were the most commonly cited barriers to applying systemic thinking usefully [11, 24, 25, 29, 39, 40, 42, 44]. Apparent conflicts with other rules and priorities were mentioned [39, 40]. Fears of the unknown and difficulty in maintaining a chain of accountability were mentioned [11, 41]. Without specific targets, some participants feared unstated institutional motives [42] or felt pointlessly imposed upon [34]. Other barriers to successful implementation were individuals’ resistance to change [24, 33, 34, 41, 44, 47], reluctance by senior staff to yield to collaborative decision-making [24, 27, 47] and lack of financial incentives [47] or external facilitation [11, 44]. Three articles provided especially detailed analyses of barriers to effective implementation [24, 44, 47].

### Time period for monitoring impacts

In a minority of studies [25, 28, 31, 38], impact monitoring occurred for less than 12 months. In some cases, the time elapsed between intervention initiation and evaluation of impacts was unclear [29, 43].

### Impacts, consequences and significance

Table 3 gives a brief list of positive impacts linked to each intervention, so is meant to be demonstrative rather than definitive about reported outcomes. Many of the measures relate to efficiency gains, only some of which directly benefit patients. In a minority of interventions [28–31, 35, 36, 43, 46], there was not a clearly described process (such as open-ended questions with participants) to facilitate recognition of negative or unintended consequences. No negative changes were linked to interventions, although some metrics failed to improve as hoped. In a multi-site trial [47–49], practices that had more contact with facilitators reported the greatest positive impacts.

Statistical significance to describe observed changes was not possible to calculate for most interventions eligible for this review, but two studies described the results of randomized clinical trials. Horbar et al. [30] reported several significantly improved indicators for speed of preterm infants receiving surfactant treatment in intervention hospitals. Parchman et al. [48] reported significantly improved (and sustained) chronic care indicators for intervention practices.

### Quality of reporting

Table 4 shows the quality assessment scores for each study. Scoring depended on details available about each intervention, which might be summarized in one or several articles. The average quality score for USA (2.4) is lower than for UK/Canada articles (3.4). The Canadian and UK studies more often reported only subjective observations about changes (such as “I have more tools which have helped when working with groups of staff” [38]). American articles mostly used objectively determined evaluation metrics (e.g. efficiency in filling out paperwork [43] or fewer patient hospitalisations [46]), but were somewhat less likely than UK/Canadian applications to look for (in evaluation methods) unintended consequences, and tended towards shorter evaluation periods. Five interventions (23 %) scored 4–5 (reported relatively well), seven had score = 3 (fair quality reporting), and ten (45 %) had scores of 2 or below (mostly due to unclear or omitted elements in reporting). Studies which were desirably informed by complexity science in their evaluation strategy (yes answers to both questions 1 and 5: reporting over time periods >1 year or looking for unintended consequences), combined with objectively measured outcomes (question 3), were in a small minority (3/22 studies, 13.6 %) [11, 23, 24, 44].

**Table 4:** Quality assessment for intervention reports

Intervention/authors	Country	Q1	Q2	Q3	Q4	Q5	Total score
Chin et al. [38]	UK	−1	1	−1	1	1	1
Dattée et al. [39]	UK	1	1	−1	1	1	3
Kothari et al. [40]	Canada	1	1	−1	1	1	3
Mowles et al. [11]	UK	1	1	1	1	1	5
Rowe et al. [41]	UK	1	1	0	1	1	4
Solomon et al. [42]	Canada	1	1	−1	1	1	3
Zimmerman et al. [24], Gardam et al. [66], Gitterman et al. [23]	Canada	1	1	1	1	1	5
Backer et al. [44]	USA	1	1	1	1	1	5
Balasubramanian et al. [27], Stroebel et al. [67]	USA	1	0	0	0	1	2
Boustani et al. [46]	USA	−1	1	1	1	−1	1
Cabin Creek [35]	USA	0	1	1	1	−1	2
Capuano et al. [28]	USA	−1	1	1	1	−1	1
Clancy et al. [43]	USA	0	1	1	1	−1	2
Fontanesi et al. [29]	USA	0	1	1	1	−1	2
Horbar et al. [30]	USA	1	1	1	1	−1	3
Kegler et al. [25]	USA	−1	1	1	−1	0	0
Khan et al. [31]	USA	−1	1	1	1	−1	1
MacKenzie et al. [32]	USA	1	1	1	1	−1	3
Moody-Thomas et al. [33, 34]	USA	1	1	1	0	−1	2
Parchman et al. [48, 49], Noël et al. [47]	USA	1	1	1	1	0	4
Positive Deviance [36, 37]	USA	1	1	1	1	−1	3
Solberg et al. [45]	USA	1	1	1	1	−1	3

*Notes*: Exact text of questions is listed in Table 2. Scores: 1 = yes, 0 = partly/unclear, −1 = No

## Discussion

Twenty-two interventions were found that stated that CS was part of the conceptual framework for design. The most common approach was to use workshops for health care professionals and managers to encourage systemic thinking to address either specific or non-specific problems. The linked positive benefits from these interventions were diverse, ranging from subjective perceptions of improved confidence in multiple situations, to specific patient benefits (such as reduced waiting times).

From more than 5000 scientific articles, only 22 eligible interventions were found that had been informed by CS and reported specific outcomes. This seems somewhat surprising, given the many very influential articles [1–8] (>250 citations each on Google Scholar, as of 1 January 2016), that endorsed complexity science for health care system improvement. Difficulty in finding suitable articles was partly due to reporting quality. A previous search for and review of CS-informed interventions in low-middle income countries also reported frustration with lack of specificity about the conceptual framework used in intervention design [9]. One of our selected interventions was not described as informed by CS in actual evaluation reports [47, 48], even though CS was cited heavily as part of the conceptual framework in the intervention protocol [49]. Another study [50] was excluded in spite of being lauded for high compatibility with CS [51], because there was no evidence that the intervention was designed with CS as part of the conceptual framework. We conclude that a comprehensive inventory of CS-informed interventions (which include evaluation) is not currently feasible. Other authors responded to this difficulty by applying less demanding evaluation evidence, and also quality grading criteria to identify CS-compatible intervention approaches, but that approach also has shortcomings if intended to form an evidence base (see “Limitations” discussion below). It may be argued that the difficulties this review found in evidence search arise because complexity theory is primarily an explanatory tool and is poorly suited for supporting intervention design [52, 53] or simply as a helpful tool in identifying potential leverage points for change. Addressing those perspectives is beyond the scope of this paper but has been discussed by others [54].

Missing mention of unintended (possibly negative) consequences in reports may suggest a misunderstanding about inherent characteristics of complex systems; unintended consequences following complex system leverage should always be expected and looked for. Process evaluation was not thorough in many of the selected studies; for example, many studies described no barriers to implementation. Hence, although CS may have informed design, it often did not inform evaluation. However, some discussions of barriers were very thorough [44, 47] and may help others to design more effective CS-informed interventions.

Barriers to implementation where they were mentioned: UK and Canadian interventions more often mentioned institutional barriers, institutional resistance to change and conflicting performance targets. This may reflect an advantage of the fragmented American health care system: it may be a more agile environment and therefore more likely to facilitate innovation. Simple and specific goals in US programmes also seem to have contributed to more tangible positive impacts on final reports. The UK/Canada approaches seemed more oriented towards general change by a small number of perhaps isolated agents, who as a result were more likely to report large institutional barriers. Plsek and Wilson [5] discussed institutional resistance to change and made recommendations for how complex adaptive systems theory can ideally be used to guide managers and health leaders to facilitate improved services.

### Limitations

Not eligible were interventions that taught health professionals to think through a complexity science lens (e.g. [55–57]), but without subsequent mention of change in actual practice or specific impacts. Also, excluded were initiatives to develop networks with potential to encourage systemic change, but typically, the only measured outcome was to verify the existence of the network contacts (e.g. [58]). Many such network, capacity or competence building programmes exist. We focused instead on specific, sustained or actual changes, especially in health service delivery or clinical targets. It also may be that changes in personal competencies or improvements in collaborative opportunities are often still not enough to affect systemic change, due to greater institutional forces [59] (also, see previous discussion on “Barriers”). We do not comment on whether the barriers identified are especially likely to be present when dealing with complex systems.

Defining what is or is not a CS-informed intervention is challenging. We wanted explicit statements in the research design about complexity science, but we acknowledge that diverse terminology might be valid. So inevitably, our eligibility criteria (see “Methods” section) cannot be perfect, although we tried to exclude similar or related descriptions only where other authors had made clear distinctions from complexity science. Only one investigator screened items for inclusion; this increases the risk that we may have overlooked some eligible articles. Other reviewers have quality graded (or “scored”) interventions according to their compatibility with CS principles, either according to expert opinion or a specific theoretical framework [10, 14–17, 60]. Whereas our research question was rather more functional, could we find out if, people who say they applied CS, managed to achieve useful results? So the eligibility test was not correct use of CS ideas, but rather stating that CS had influenced intervention design, with specific apparent impacts.

Therefore, we did not attempt to estimate how much intervention design was informed by CS vs. other conceptual frameworks. Inventorying the range of theoretical foundations in intervention design would be an exhaustive and probably still not definitive exercise. Moreover, we relied heavily on information that was in the abstract, title or keywords; if these did not suggest application of complexity science in intervention design, then the article was usually excluded. It would be an enormous effort to screen every article published since 1995 that mentioned any CS concept in the main text, for application of CS in real-world intervention design.

It is quite conceivable that some of the selected studies cited CS as part of the conceptual framework in research design without understanding or truly embracing many aspects of CS. Some authors have explicitly addressed possible misapplications of CS [60, 61]. Other researchers [10, 14, 15, 62] looked in interventions for CS principles, by grading items for eligibility, rather than rely on CS phrases. There is merit in evaluating compatibility with CS, but also drawbacks. It is to be expected, in empirical efforts, that implementors adopt and adapt ideas for our own purposes. It can even be argued that applying CS principles flexibly (picking and choosing) is, in fact, applying CS ideas correctly. This applies even in the context of research design, which otherwise, traditionally, is developed by rigid (“clockware”) methods [63]. It may be argued that using CS in the real-world means, ideally, an agile and adaptive process [64, 65]. Therefore, quality grading or rating interventions that said they were informed by CS, to try to calculate how much they were truly informed by CS, could be antithetical to the spirit of how CS is supposed to be applied to address real-life problems.

### Recommendations

This scoping review leads to a number of suggestions how to improve future implementation and reporting of CS-informed interventions and to make evidence more available and useful for adaptation by others. Many of these principles apply to all public health interventions and indeed to product development and other types of projects. Complexity science or related phrases should be in article titles, abstracts or keywords. Interventions with specific simple targets at project inception that can be measured objectively seem to report more tangible outcomes—that are also more compatible with evidence-based medicine. Results should be monitored for a minimum of 12 months. Barriers to implementation need to be explored and discussed, to help others foresee and perhaps mitigate in own applications. Unintended or negative changes should be actively looked for. Perhaps, most importantly, success seems to depend on support by institutions and senior staff combined with widespread collaborative effort. Applying CS principles to effect system change seems especially unlikely to produce significant success if led or imposed by single individuals or indeed if undertaken by isolated individuals; rather, CS ideas may be the most effective as impetus for change when applied as part of a process involving many stakeholders who perceive that they equally own the process towards change and who have institutional support to prioritize this process over other institutional targets. Nonetheless, facilitation by nominated individuals or external incentives can also be beneficial.

In the short and medium term, investigators looking for evidence that complexity-informed interventions are effective will probably continue to need to seek theoretical and CS-compatible studies rather than expect to find clearly documented evidence of positive impacts from embracing complexity-informed conceptual frameworks.

## Conclusions

Intervention elements that are compatible with CS are common; but these elements may be compatible with or indeed inspired by other theoretical frameworks, too. Moreover, many articles about CS-compatible interventions do not discuss intervention design in the context of CS. This makes it difficult to find evidence that using “the lens” of complexity science is actually useful in intervention design or implementation.

It is not yet feasible to confidently evaluate the efficacy of complexity-informed interventions. It is not unusual that an intervention is lauded for including many core principles of CS, even though the research report itself never mentions CS or CS-related concepts as part of the conceptual framework underpinning design. Meanwhile, other researchers apply methods without realizing that their intervention approach only came to prominence because of endorsement by others for being compatible with CS principles. Even when an intervention clearly states it was designed using CS, there may be no indication that CS was used in evaluation strategy. It is thus clear that many reports on CS-informed interventions give incomplete information about aspects of applying CS that are integral to understanding elements of successful implementation—e.g. information is missing about barriers to implementation, contextual factors or unintended or negative consequences. These last practical insights could make CS-informed interventions (indeed all interventions) much easier for others to adapt and make effective.

We cannot and indeed should not expect everyone to explain all their interventions according to theory. Nevertheless, the endorsement of CS thinking to improve health care delivery is undermined by lack of empirical evidence [2, 12]. People will be less inclined to apply CS to design improvements in health care without evidence that it works [13]. Some argue that cause and effect are inherently impossible to establish in CAS, but it seems (ironically and perhaps suitably), that even finding CS-informed interventions is in itself, a complex problem.

A number of recommendations are listed to make reporting and details about implementation of CS-informed interventions easier to find and useful to other researchers—to make CS-informed intervention design and implementation work in the real world.

## Additional files

Additional file 1: Search strategy for finding potentially eligible studies. (DOCX 37 kb)

Additional file 2: Extracted data on intervention format, complexity science concepts as described by authors, timescale for observations, target conditions or problems, and specific positive outcomes linked to each intervention. (DOCX 58 kb)

## Abbreviations

CS: Complexity science, complex systems or complex adaptive systems
UK: United Kingdom
USA: United States of America
