# Supplementary material for: Do complexity-informed health interventions work? A scoping review
Source: Implement Sci. 2016 Sep 20;11:127. doi: 10.1186/s13012-016-0492-5 (PMC5029105; doi:10.1186/s13012-016-0492-5)
Supplement: Supplementary file 1 — Search strategy for finding potentially eligible studies. (DOCX 37 kb) [file 13012_2016_492_MOESM1_ESM.docx]

**SECTION 1. Scientific literature search phrases / strategy**

The search strategy tried to achieve a balance between specificity & sensitivity

OVID (Amed, Embase, Medline)

1. health.tw.

2. (complex-adaptive-syste$ or systems-thinking or complex-health-syste$ or complexity-science).tw.

3. (complex-system$ or syste$-change$).ti.

4. (complex-causal-pathway$ or complex-systemic).tw.

5. complexity.ti. and system$.tw.

6. 1 and (2 or 3 or 4 or 5)

7. limit 6 to yr=1995-current

8. limit 7 to humans

9. remove duplicates from 8

SCOPUS (only health sciences database), tw = title words

Health(tw) and (tw) complex-adaptive-systems or systems-thinking or complex-health systems or complexity-science or complex-systemic -> 1026

OR

Health(tw) and (complexity(ti) and system.tw) -> 577

OR

Health(tw) and (complex-systems or system-change).ti. -> 287

OR

Health(tw) and (complex-causal-pathway or complex-systemic).tw. -> 29

Unduplicated total = 1919

**Calibrated** by making sure the search found these 4 (chosen from preliminary literature searches as specific examples of evaluated interventions undertaken with complex-systems/analogous thinking in mind)

Mutale et al 2013 [Application of systems thinking: 12-month postintervention evaluation of a complex health system intervention in **Zambia**: the case of the **BHOMA**.](http://www.ncbi.nlm.nih.gov/pubmed/26011652)

Prashanth et al 2014 Advancing the application of systems thinking in health: a realist evaluation of a capacity building programme for district managers in Tumkur, India

Moody-thomas et al 2015 [Effect of systems change and use of electronic health records on quit rates among tobacco users in a public hospital system](http://www.scopus.com/record/display.uri?eid=2-s2.0-84924712098&origin=resultslist&sort=plf-f&src=s&st1=systems-change&st2=tobacco&sid=82BB2FA76F310E63A53079643CE6B233.N5T5nM1aaTEF8rE6yKCR3A%3a10&sot=b&sdt=b&sl=136&s=%28TITLE-ABS-KEY%28systems-change%29+AND+TITLE-ABS-KEY%28tobacco%29%29+AND+SUBJAREA%28MULT+OR+MEDI+OR+NURS+OR+VETE+OR+DENT+OR+HEAL%29+AND+PUBYEAR+%3e+1999&relpos=1&citeCnt=0&searchTerm=%28TITLE-ABS-KEY%28systems-change%29+AND+TITLE-ABS-KEY%28tobacco%29%29+AND+SUBJAREA%28MULT+OR+MEDI+OR+NURS+OR+VETE+OR+DENT+OR+HEAL%29+AND+PUBYEAR+%26gt%3B+1999)

Moody-Thomas et al 2013 Use of systems change and health information technology to integrate comprehensive tobacco cessation services in a statewide system for delivery of healthcare

**SECTION 2.** **Grey Literature database searches.**

The first 20 hits were screened from the total found by searching given source with the phrase (developed after screening the peer-review literature).

Specific search phrase, using Google search engine where own website search engine not used or available, unless otherwise noted, was

"complex systems" health intervention evaluation systemic complexity application implementation "case study".

| **Source** | **Gross No. of Results (**only 1^st^ 40 screened) | **No. of new reports included in review** |
| --- | --- | --- |
| *Searched using Google search (engine) algorithm* |  |  |
| Open Google (free text) search | 388,000 | 2 [[1](#_ENREF_1), [2](#_ENREF_2)] |
| www.cdc.gov | 15 | 0 |
| www.ntis.gov  (phrase = complex health systems) | 19 | 0 |
| www.docuticker.com  (phrase = complex health systems) | 1 | 0 |
| www.who.int | 21,400 | 0 |
| www.msf.org  (phrase = complex health systems) | 606 | 0 |
|  |  |  |
| *Searched using website’s own search engine* |  |  |
| www.apa.org  (phrase = complex health systems) | 1052 | 0 |
| greylit.org, words in title or summary contain  (complex* + health) or (complex* + health + systems) or (complex* theory) | 16 | 0 |
| www.opengrey.eu  ("complex systems" health) or (complexity health systems) | 45 | 0 |
| www.who.int  (phrase = complex health systems) | 4450 | 0 |

**SECTION 3. Supplemental searches (3 approaches)**

Plexus website (“case study”, using Google search engine): https://plexusinstitute.site-ym.com/. Two inclusions eligible for full text review [[3](#_ENREF_3), [4](#_ENREF_4)].

“public health case study” search phrase for journal *Systematic Practice and Action Research*

Snowballing (hand searching references of) other inventories [[5-8](#_ENREF_5)] and included studies: Six new inclusions suitable for full text review [[9-14](#_ENREF_9)].

**REFERENCES**

1. Capuano T, MacKenzie R, Pintar K, Halkins D, Nester B: **Complex adaptive strategy to produce capacity-dfiven financial improvement**. *J Healthc Manag* 2009, **54**(5):307.

2. Dattée B, Barlow J: **Complexity and whole-system change programmes**. *J Health Serv Res Policy* 2010, **15**(2):19-25.

3. Plexus Institute: **Cabin Creek: Organizational Change: Improved Pain Management and Better Rural Healthcare**; 2015.

4. **Positive Deviance in MRSA prevention** [<http://www.plexusinstitute.org/?page=healthquality>]

5. Leykum LK, Pugh J, Lawrence V, Parchman M, Noël PH, Cornell J, McDaniel RR: **Organizational interventions employing principles of complexity science have improved outcomes for patients with Type II diabetes**. *Implementation Science* 2007, **2**(1):28.

6. Riley BL, Robinson KL, Gamble J, Finegood DT, Sheppard D, Penney TL, Best A: **Knowledge to action for solving complex problems: Insights from a review of nine international cases**. *Chronic Dis Inj Can* 2015, **35**(3):47-53.

7. Holmes BJ, Finegood DT, Riley BL, Best A: **Systems Thinking in Dissemination and Implementation Research**. In: *Dissemination and Implementation Research in Health.* edn. Edited by Brownson RC, Colditz GA, Proctor EK: Oxford University Press; 2012: 560.

8. Lich KH, Ginexi EM, Osgood ND, Mabry PL: **A call to address complexity in prevention science research**. *Prevention science* 2013, **14**(3):279-289.

9. Parchman ML, Noel PH, Culler SD, Lanham HJ, Leykum LK, Romero RL, Palmer RF: **A randomized trial of practice facilitation to improve the delivery of chronic illness care in primary care: initial and sustained effects**. *Implemention Science* 2013, **8**(93):1-7.

10. Zimmerman B, Reason P, Rykert L, Gitterman L, Christian J, Gardam M: **Front-line ownership: Generating a cure mindset for patient safety**. *Healthcare Papers* 2013, **13**(1):18.

11. Gardam M, Reason P, Gitterman L: **Healthcare-associated infections: New initiatives and continuing challenges**. *Healthc Q* 2012, **15**:36-41.

12. Lindberg C, Herzog A, Merry M, Goldstein J: **Health care applications of complexity science. Life at the edge of chaos**. *Physician Exec* 1998, **24**(1):6-20.

13. Moody-Thomas S, Nasuti L, Yi Y, Celestin M.D, Jr., Horswell R, Land TG: **Effect of systems change and use of electronic health records on quit rates among tobacco users in a public hospital system**. *Am J Public Health* 2015, **105**:e1-e7.

14. Noël PH, Romero RL, Robertson M, Parchman ML: **Key activities used by community based primary care practices to improve the quality of diabetes care in response to practice facilitation**. *Qual Prim Care* 2014, **22**(4):211-219.
