# Supplementary material for: Do complexity-informed health interventions work? A scoping review
Source: Implement Sci. 2016 Sep 20;11:127. doi: 10.1186/s13012-016-0492-5 (PMC5029105; doi:10.1186/s13012-016-0492-5)
Supplement: Supplementary file 2 — Extracted data on intervention format, complexity science concepts as described by authors, timescale for observations, target conditions or problems, and specific positive outcomes linked to each intervention. (DOCX 58 kb) [file 13012_2016_492_MOESM2_ESM.docx]

**Evidence Table**

| **Study, where** | **Description of Intervention and Comparator (if any)** | **How the article(s) stated that complexity science or very similar concepts informed the intervention design** | **What health condition, care procedure or system was targeted** | **Outcomes/Changes attributed to the intervention** | **Maximum duration of obs.** |
| --- | --- | --- | --- | --- | --- |
| *UK and Canada studies* | | | | | |
| Chin et al. 2006 [[1](#_ENREF_1)], UK | 7 days of workshops ran over a 6 month period to encourage more collaborative ways of working.  Pre/post comparisons | (Background) [This] “model embraces and recognizes the complexity and messiness of practice development by adopting a whole systems approach to enabling it.” | Improvement in patient care and services | * Confidence about future improvements in working practice  * More tools when working with groups of staff  * Considering the bigger picture along with the little picture  * Taking time to plan and think is now integral part of leadership style  * More proposals to improve working practices put forward by staff  * More stress on evidence-based practice, more support for practice changes  * Better relations with colleagues  * More effective and seen as more effective | 6 months |
| Dattée et al. 2010 [[2](#_ENREF_2)], UK | The Unscheduled Care Collaborative Programme was set up to help deliver performance targets for emergency care: to provide an overall structure for quick trial (and discard if appropriate) of actions that had seemed to improve services elsewhere. Implemented through a series of workshops with health care and management staff.  Pre/post comparisons | (Objectives) Applying complexity theory to effect health care systems change, and to evaluate results of such an initiative | System performance metrics | * Change in mindset from blame to mutual effort by managers  * Flexibility allowed by programme design encouraged local innovation  * Empowered local flow leaders to work around departmental interdependencies and successfully overcome underlying resistance | 22 months |
| Kothari et al. 2015 [[3](#_ENREF_3)], Canada | Knowledge translation initiative, implemented as Webinar series for community groups  Pre/post comparisons | (Background) [stated intention to] engage communities of practice within a complex system | Generic health system support | *Participants indicated that they were part of a positive collaboration | 24 months |
| Mowles et al. 2010 [[4](#_ENREF_4)], UK | External training, audits, surveys and system reviews  Pre/post comparisons | …“an approach based on theories of complexity”: Drew on organisational development and theories of complexity, presented here as complex adaptive systems theory. [These] are also variations of a systemic understanding of change. | Therapy department in Scotland for people with disabilities | * A significant decrease in the level of complaints about the service  * significant reduction of waiting times across the child and adult services  * Managers reported enhanced ability to discuss, share and decide upon both shorter and longer term organisational problems, some of which were intractable  * More confident and less defensive attitude on the part of staff and a clearer understanding of how the service needed to develop  * Better focus on one area for a significant length of time  * Freedom to get away from looking for solutions and how to “fix” issues – although some solutions have emerged along the way | 24 months |
| Rowe et al. 2005 [[5](#_ENREF_5)], UK | Series of workshops & audits to encourage changes in working practices  Pre/post comparisons | (Methods) “Change was brought about through use of a Complex Adaptive Systems Approach” | Primary care services | * Increase in population-based rather than individually focused work  * Increased focus on equality  * Formation of new working relationships  * Flexibility to work from and with the agenda of local communities responding creatively to areas of need | 18 months |
| Solomon et al. 2014 [[6](#_ENREF_6)], Canada | Education programme to promote more collaborative working practices (workshops)  Pre/post comparisons | (Methods) complexity-informed educational approach | Community agencies involved in care of disabled children | * Quicker, more concise and appropriate referrals  * More confidence about referring clients | 15 months |
| Zimmerman et al. 2013 [[7](#_ENREF_7)], Gardam et al. 2012 [[8](#_ENREF_8)], Gitterman et al. 2013 [[9](#_ENREF_9)], Canada | Workshops to change staff working practices and attitudes towards problem ownership, also to encourage positive deviance  Pre/post comparisons | Complexity science informed / based/ approach | Hospital acquired infections | * hand hygiene compliance increase (41% in 2008/2009, 88% in the first quarter of 2012/2013)  * over a similar time period rates of Clostridium difficile-associated disease (CDAD) decreased (0.58 cases per 1,000 patient days in 2007/2008, 0.46 cases per 1,000 patient days in the first quarter of 2012/2013)  * Rates of MRSA also decreased across the organization (0.41 cases per 1,000 patient days in 2007/2008, 0.33 cases per 1,000 patient days in 2011/2012).  * Staff from all disciplines now exhibit a greater sense of ownership with respect to hand hygiene, which has the potential to lead to sustainable change  * subsequent outbreak was recognised faster & responded to more effectively | 1.5 – 4 years (varies by setting) |
|  | *USA studies* |  |  |  |  |
| Backer et al. 2005 [[10](#_ENREF_10)] | Feedback sessions to make staff aware of system and patient barriers to uptake of screening tests, and to encourage staff to identify and redress other system barriers  Pre/post comparisons | (Background) Statement that change strategies must recognise [primary care] practices as complex systems that require complex strategies to sustain change. | Cervical smear and mammogram screening rates, along with related working practices | * Better identification and targeting of patients  * Small increases (not statistically significant) in some practices in mammogram and cervical smear rates | 12 months |
| Balasubramanian et al. 2010 [[11](#_ENREF_11)], Stroebel et al. 2005 [[12](#_ENREF_12)] | Team meetings to identify and act upon problems, 1 hour each over 12 weeks  Pre/post comparisons | Meeting structure was  informed by complexity science | Adherence to clinical guidelines | * Improvements in communication  * Faster completion of prescription refills | 2 years |
| Boustani et al. 2011 [[13](#_ENREF_13)] | Seminars and meetings with clinicians explaining communication strategy for them to follow.  Pre/post comparison | “Using the lens of the complex adaptive system and the five principles of the reflective adaptive process (Stroebel et al., 2005 [[12](#_ENREF_12)]), we implemented [a] local collaborative dementia care model.” | Dementia care | * Reduced total number of visits  * More (relevant) tests and procedures undertaken  * Shorter median /mean hospital stay  * Fewer hospitalisations  * Reduced patients with 1+ emergency dept. visits  * Lower % of patients rehospitalised within 30 days of discharge  * Fewer anticholinergic drugs  * More medication for dementia & depression | 12 months |
| Cabin Creek 2015 [[14](#_ENREF_14)] | Workshop with health care professionals and managers  Pre/post comparison | Complexity-informed process to promote system change | Pain management medication | * Physicians concerned about overuse of narcotic pain medications found 400 patients with narcotic prescriptions at one clinic alone. After 18 months of work with patients and staff only 30 patients there had such prescriptions | Unclear, but minimum 18 months |
| Capuano et al. 2009 [[15](#_ENREF_15)] | Meetings and innovation encouraged to identify bottleneck points and rapid trial/rapid failure testing of potential solutions  Pre/post comparison | (Executive summary): An efficiency model based on the complex adaptive systems approach of “swarmware” | Hospital wide admissions capacity | * Length of stay was decreased to 0.1 days below budget in the 60-day time frame  * All admissions and potential admissions were accepted (saying "yes" to patients) through key points of access in the hospital  * Transfer Center denials were reduced to 0 in 19 days  * Monthly ED diversions decreased from 110 hours to 20 hours in 60 days | 60 days |
| Clancy et al. 2006 [[16](#_ENREF_16)] | Simplication of language in patient care forms by improvement team composed of nursing staff  Pre/post comparison | (Background) Complex adaptive systems, expected power law trade off (80% of cases fall into 20% of diagnoses, and inverse is also true) | In hospital nursing care | * High satisfaction with the change by users (nurses)  * Strong agreement that new forms easier to use  * 64% calculated increase in efficiency (paperwork)  * Significant paper savings, 9 sheets per patient | Unclear |
| Fontanesi et al. 2014 [[17](#_ENREF_17)] | Reorganisation of appointment time allocation by teams with patient-centred care mission statement and probabilistic scheduling schema, and with encouragement of rapid trial/failure innovations in finding solutions to local needs  Pre/post comparison | (Title) Quality improvement for a recognised complex adaptive system | Appointment and referral management for patients with chronic liver disease | * 25% increase in volume, representing a total of 9500 visits.  * wait times for next available appointments were reduced from five months to two weeks  * unscheduled appointment slots dropped from 7% to less than 1%  * no-show” rates dropped to less than 10%  * 40% reduction in patient wait and staff idle time  * Four-fold increase in new referrals | Unclear |
| Horbar et al. 2004 [[18](#_ENREF_18)] | Intervention: Audit, feedback, evidence review, interactive training workshop, ongoing faculty support in form of conference calls and emails.  Control: Received centre-specific, confidential reports routinely prepared for members of the Vermont Oxford Network [[19](#_ENREF_19)].  Randomised Controlled Trial | Multifaceted collaborative quality improvement intervention based on promoting four key habits, including systems thinking | Delivery of lung surfacent to preterm infants | * infants in intervention hospitals were more likely to receive surfactant in the delivery room (adjusted odds ratio 5.38 (95% confidence interval 2.84 to 10.20))  * were less likely to receive the first dose more than two hours after birth (adjusted odds ratio 0.35 (0.24 to 0.53))  * received the first dose of surfactant sooner after birth (median of 21 minutes v 78 minutes, P < 0.001) | Trial ran for > 1 yr but individual obs. made within 3 days |
| Kegler et al. 2007 [[20](#_ENREF_20)] | 4 day workshop on working practices  Pre/post comparison | Part of curriculum covered systems thinking | Faith-based collaborations with public health initiatives | * Gains in leadership skills  * Better time management by individuals  * Different perspectives on conflict  * Better empathy for colleagues, better listening  * Improved collaboration with other groups  * Better team planning and dynamics | 8 months |
| Khan et al. 2015 [[21](#_ENREF_21)] | Collaborative care team operating in a regular patient service clinic, operating under patient-centred objectives, assessing needs from a questionnaire on broad-spectrum patient-centred outcomes  Pre/post comparison | (Theoretical Foundations) Collaborative care model developed using principles of complex adaptive systems from Stroebel et al. 2005 [[12](#_ENREF_12)] | Patient recovery after discharge from intensive care unit | * Reduction in the average total HABC-M (brain function impediment) score, which dropped from 19.21 points at visit 1 to 14.75 points at visit 2  * Score improvements were seen in all domains as well as in the total score, and were significant for all but the behavioural-psychological domain | 6 months for individual patients, duration entire programme = 10 months |
| MacKenzie et al. 2008 [[22](#_ENREF_22)] | Staff retreat to teach all to think systematically, improvement team and procedures set up to try rapid testing/failure of possible innovations and solutions  Pre/post comparison | Complex systems, complex adaptive systems | Organisational capacity (in hospital admissions) | * Admissions rose by 6.1%, 5.5%, 8.7%, 5.0%, and 3.8% in fiscal years 2003 through 2007  * Process enhancements cost approximately $1 million, while increased revenues attributable to increased capacity totalled $2.5 million  * Transfer centre requests increased 4 times from 2003-2007, while acceptance rate > 95% was maintained  * Turnaround time for beds reduced from 210 minutes to 54 minutes in 2004, and was maintained (to 2007)  * Time for bed cleaned to bed occupied by next pt decreased by 40 minutes, 65% decrease  * ED visits per bed increased 21-30% at 2 hospital locations  * OR holds (shut downs) decreased from 750 minutes to less than 125 minutes/month | 24 months |
| Moody-Thomas  et al. 2013 & 2015 [[23](#_ENREF_23), [24](#_ENREF_24)] | Information and systematic organisation to facilitate identification of patients who smoke, and targeting of cessation services at them.  Pre/post comparison | "Systems thinking was used as the framework for [the tobacco control programme] conceptualization and development." | Smoking cessation services | * The number of patients reporting tobacco use in the past 30 days decreased among the population who had at least 1 primary care visit in the first 6 months of the study period  * 9.5% relative decrease in prevalence [of tobacco use in preceding 30 days] was significant (P < .001).  * Quit rates increased from 5.0% in 2008 to 9.6% in 2009  * Chronically ill patients (except those with HIV) reported lower 30-day (26%) prevalence rates than the general patient population  * record keeping & patient identification became more efficient | 3 years |
| Parchman et al. 2008 & 2013 [[25](#_ENREF_25), [26](#_ENREF_26)], Noël et al. 2014 [[27](#_ENREF_27)] | Intervention: Practice faciliators (PF) held 6+ meetings with practice staff over 1 year, to promote interactive consensus building and goal setting, for management of patients who had type 2 diabetes.  Control: Waiting list for 1 year.  Group randomised trial, and pre/post comparisons | Protocol states that the PF approach is a way to “account for the complex adaptive system (CAS) characteristics of the primary care setting.” | Type 2 diabetes case management | * There was significant improvement in [chronic care] scores (p < 0.05) within initial intervention practices, from 5.58 (SD 1.89) to 6.33 (SD 1.50), compared to the delayed intervention (control) practices where there was a small decline, from 5.56 (SD 1.54) to 5.27 (SD 1.62).  * Scores for chronic care were sustained at the improved level for one year after intervention end, while scores in the delayed intervention (control) practices improved during their one year of intervention, from 5.27 (SD 1.62) to 5.99 (SD 1.75). | 3 years |
| Positive Deviance 2010-2015 [[28](#_ENREF_28), [29](#_ENREF_29)] | Group sessions and staff education to promote systemic thinking, positive changes and successful strategies | Informed by complex systems theory | Hospital acquired (HA) infections | * Institution 1: A 70% drop in the rate of HA MRSA infections from 2006 to 2008, yielding a 2008 rate of 0.81 infections/ 1000 patient days.  * Institution 2: 100% drop in the rate of HA MRSA infections from 2006 to 2008, yielding a 2008 rate of 0.00 infections/1000 patient days.  * Institution 3: 65% drop in the rate of HA MRSA infections from 2006 to 2008, yielding a 2008 rate of 4.33 infections/1000 patient days. | 3 years |
| Solberg et al. 2007 [[30](#_ENREF_30)] | Working with a mentor, clinical supervisor led regular staff meetings to encourage staff to identify and rapid trial better working practices | (Title, abstract, introduction) Systems thinking and systems approach | Care for patients with T2 diabetes | * Quarterly measures [composite diabetes management scores] rose from 5.7% to 42.9%, while the 7000 diabetes patients of the entire medical group only increased from 4.2% to 12.1%.  * Colorectal cancer screening and tobacco advice rates (also) rose by 50% | 2 years |

Notes: Evidence Table: obs = observations, SD = standard deviation.

**REFERENCES**

1. Chin H, Hamer S: **Enabling practice development: Evaluation of a pilot programme to effect integrated and organic approaches to practice development**. *Practice Development in Health Care* 2006, **5**(3):126-144.

2. Dattée B, Barlow J: **Complexity and whole-system change programmes**. *J Health Serv Res Policy* 2010, **15**(2):19-25.

3. Kothari A, Boyko JA, Conklin J, Stolee P, Sibbald SL: **Communities of practice for supporting health systems change: A missed opportunity**. *Health Research Policy and Systems* 2015, **13**(1).

4. Mowles C, van der Gaag A, Fox J: **The practice of complexity: Review, change and service improvement in an NHS department**. *Journal of Health, Organisation and Management* 2010, **24**(2):127-144.

5. Rowe A, Hogarth A: **Use of complex adaptive systems metaphor to achieve professional and organizational change**. *J Adv Nurs* 2005, **51**(4):396-405.

6. Solomon P, Risdon C: **A process oriented approach to promoting collaborative practice: Incorporating complexity methods**. *Med Teach* 2014, **36**(9):821-824.

7. Zimmerman B, Reason P, Rykert L, Gitterman L, Christian J, Gardam M: **Front-line ownership: Generating a cure mindset for patient safety**. *Healthcare Papers* 2013, **13**(1):18.

8. Gardam M, Reason P, Gitterman L: **Healthcare-associated infections: New initiatives and continuing challenges**. *Healthc Q* 2012, **15**:36-41.

9. Gitterman L, Reason P, Gardam M: **Front line ownership approach to improve hand hygiene compliance and reduce health care-associated infections in a large acute care organization**. *Canadian Journal of Infectious Diseases and Medical Microbiology* 2013, **24**:21B-22B.

10. Backer EL, Geske JA, McIlvain HE, Dodendorf DM, Minier WC: **Improving female preventive health care delivery through practice change: An every woman matters study**. *J Am Board Fam Pract* 2005, **18**(5):401-408.

11. Balasubramanian BA, Chase SM, Nutting PA, Cohen DJ, Strickland PAO, Crosson JC, Miller WL, Crabtree BF, Team US: **Using learning teams for reflective adaptation (ULTRA): Insights from a team-based change management strategy in primary care**. *The Annals of Family Medicine* 2010, **8**(5):425-432.

12. Stroebel CK, McDaniel RR, Crabtree BF, Miller WL, Nutting PA, Stange KC: **How complexity science can inform a reflective process for improvement in primary care practices**. *Joint Commission Journal on Quality and Patient Safety* 2005, **31**(8):438-446.

13. Boustani MA, Sachs GA, Alder CA, Munger S, Schubert CC, Guerriero Austrom M, Hake AM, Unverzagt FW, Farlow M, Matthews BR *et al*: **Implementing innovative models of dementia care: The healthy aging brain center**. *Aging and Mental Health* 2011, **15**(1):13-22.

14. Plexus Institute: **Cabin Creek: Organizational Change: Improved Pain Management and Better Rural Healthcare**; 2015.

15. Capuano T, MacKenzie R, Pintar K, Halkins D, Nester B: **Complex adaptive strategy to produce capacity-dfiven financial improvement**. *J Healthc Manag* 2009, **54**(5):307.

16. Clancy TR, Delaney CW, Morrison B, Gunn JK: **The benefits of standardized nursing languages in complex adaptive systems such as hospitals**. *J Nurs Adm* 2006, **36**(9):426-434.

17. Fontanesi J, Martinez A, Boyo T, Gish R: **A case study of quality improvement methods for complex adaptive systems applied to an academic hepatology program**. *The Journal of Medical Practice Management* 2014, **30**(5):323-327.

18. Horbar JD, Carpenter JH, Buzas J, Soll RF, Suresh G, Bracken MB, Leviton LC, Plsek PE, Sinclair JC: **Collaborative quality improvement to promote evidence based surfactant for preterm infants: A cluster randomised trial**. *Br Med J* 2004, **329**(7473):1004.

19. Horbar JD: **The Vermont Oxford Network: evidence-based quality improvement for neonatology**. *Pediatrics* 1999, **103**(Supplement E1):350-359.

20. Kegler MC, Kiser M, Hall S: **Evaluation findings from the Institute for Public Health and faith collaborations**. *Public Health Rep* 2007, **122**(6):793-802.

21. Khan BA, Lasiter S, Boustani MA: **Critical care recovery center: An innovative collaborative care model for ICU survivors**. *Am J Nurs* 2015, **115**(3):24-31.

22. MacKenzie R, Capuano T, Durishin LD, Stern G, Burke JB: **Growing organizational capacity through a systems approach: One health network's experience**. *Joint Commission Journal on Quality and Patient Safety* 2008, **34**(2):63-73.

23. Moody-Thomas S, Celestin Jr MD, Horswell R: **Use of systems change and health information technology to integrate comprehensive tobacco cessation services in a statewide system for delivery of healthcare**. *Open J Prev Med* 2013, **3**(01):75.

24. Moody-Thomas S, Nasuti L, Yi Y, Celestin M.D, Jr., Horswell R, Land TG: **Effect of systems change and use of electronic health records on quit rates among tobacco users in a public hospital system**. *Am J Public Health* 2015, **105**:e1-e7.

25. Parchman ML, Noel PH, Culler SD, Lanham HJ, Leykum LK, Romero RL, Palmer RF: **A randomized trial of practice facilitation to improve the delivery of chronic illness care in primary care: initial and sustained effects**. *Implemention Science* 2013, **8**(93):1-7.

26. Parchman ML, Pugh JA, Culler SD, Noel PH, Arar NH, Romero RL, Palmer RF: **A group randomized trial of a complexity-based organizational intervention to improve risk factors for diabetes complications in primary care settings: study protocol**. *Implemention Science* 2008, **3**(1):15.

27. Noël PH, Romero RL, Robertson M, Parchman ML: **Key activities used by community based primary care practices to improve the quality of diabetes care in response to practice facilitation**. *Qual Prim Care* 2014, **22**(4):211-219.

28. **Positive Deviance in MRSA prevention** [<http://www.plexusinstitute.org/?page=healthquality>]

29. Lindberg C, Clancy TR: **Positive deviance: an elegant solution to a complex problem**. *J Nurs Adm* 2010, **40**(4):150-153.

30. Solberg LI, Klevan DH, Asche SE: **Crossing the quality chasm for diabetes care: The power of one physician, his team, and systems thinking**. *J Am Board Fam Med* 2007, **20**(3):299-306.
